# Mortality Benefit of a Fourth-Generation Synchronous Telehealth Program for the Management of Chronic Cardiovascular Disease: A Longitudinal Study

**DOI:** 10.2196/jmir.5718

**Published:** 2016-05-13

**Authors:** Chi-Sheng Hung, Jiun-Yu Yu, Yen-Hung Lin, Ying-Hsien Chen, Ching-Chang Huang, Jen-Kuang Lee, Pao-Yu Chuang, Yi-Lwun Ho, Ming-Fong Chen

**Affiliations:** ^1^Telehealth CenterNational Taiwan University HospitalTaipeiTaiwan; ^2^Department of Business AdministrationCollege of ManagementNational Taiwan UniversityTaipeiTaiwan; ^3^Division of CardiologyDepartment of Internal MedicineNational Taiwan University HospitalTaipeiTaiwan; ^4^Department of NursingNational Taiwan University HospitalTaipeiTaiwan

**Keywords:** cardiovascular diseases, telemedicine, all-cause mortality, outcome assessment (health care)

## Abstract

**Background:**

We have shown that a fourth-generation telehealth program that analyzes and responds synchronously to data transferred from patients is associated with fewer hospitalizations and lower medical costs. Whether a fourth-generation telehealth program can reduce all-cause mortality has not yet been reported for patients with chronic cardiovascular disease.

**Objective:**

We conducted a clinical epidemiology study retrospectively to determine whether a fourth-generation telehealth program can reduce all-cause mortality for patients with chronic cardiovascular disease.

**Methods:**

We enrolled 576 patients who had joined a telehealth program and compared them with 1178 control patients. A Cox proportional hazards model was fitted to analyze the impact of risk predictors on all-cause mortality. The model adjusted for age, sex, and chronic comorbidities.

**Results:**

There were 53 (9.3%) deaths in the telehealth group and 136 (11.54%) deaths in the control group. We found that the telehealth program violated the proportional hazards assumption by the Schoenfeld residual test. Thus, we fitted a Cox regression model with time-varying covariates. The results showed an estimated hazard ratio (HR) of 0.866 (95% CI 0.837-0.896, *P*<.001; number needed to treat at 1 year=55.6, 95% CI 43.2-75.7 based on HR of telehealth program) for the telehealth program on all-cause mortality after adjusting for age, sex, and comorbidities. The time-varying interaction term in this analysis showed that the beneficial effect of telehealth would increase over time.

**Conclusions:**

The results suggest that our fourth-generation telehealth program is associated with less all-cause mortality compared with usual care after adjusting for chronic comorbidities.

## Introduction

Cardiovascular disease (CVD) is a major health burden worldwide [1,2]. To improve the efficacy and reduce the burden on the health care system, telemonitoring technology has been applied to the disease management program for chronic CVD. The mortality benefit of the telehealth program for chronic heart failure has been demonstrated repeatedly [3,4]. Telemedicine has also been shown to improve the control of vascular risk factors among patients with established cardiovascular diseases [5]. However, the evidence for the long-term (more than 1 year) benefits of a telehealth program in other chronic cardiovascular diseases is inadequate. A randomized controlled trial of a telehealth program using a nonimmediate data analysis system for 1 year resulted in increased mortality among elderly patients with chronic diseases [6]. This result raised a serious concern about the use of telehealth programs in managing patients with chronic CVD.

Because of the technologies adopted, different telehealth care programs provide different levels of care integration. Based on the integration of care, Anker and coworkers [7] classified telehealth programs into four generations. The fourth, or newest, generation of telehealth programs provides the continuous presence of a physician and nursing staff to analyze and respond synchronously to newly acquired patient data. We have shown in our prior studies that our fourth-generation telehealth care program was associated with a lower rate of emergency department visits and hospitalizations among patients with chronic CVD [8,9]. However, the long-term impact of a fourth-generation telehealth program on mortality among patients with chronic CVD has not yet been reported in the literature.

Based on our previous reports, we hypothesized that a fourth-generation telehealth program could reduce mortality in patients with chronic CVD. To test this hypothesis, we retrospectively analyzed the all-cause mortality data among 576 patients with chronic CVD who received the telehealth care program and 1175 patients who did not receive telehealth care.

## Methods

### Study Design

This was a single-center, clinical retrospective epidemiologic study and was approved by the Institutional Review Board of National Taiwan University Hospital, Taipei, Taiwan. All experiments in this study were performed in accordance with relevant guidelines and regulations. Informed consent was obtained from all participants.

### Recruitment

**Figure 1:**
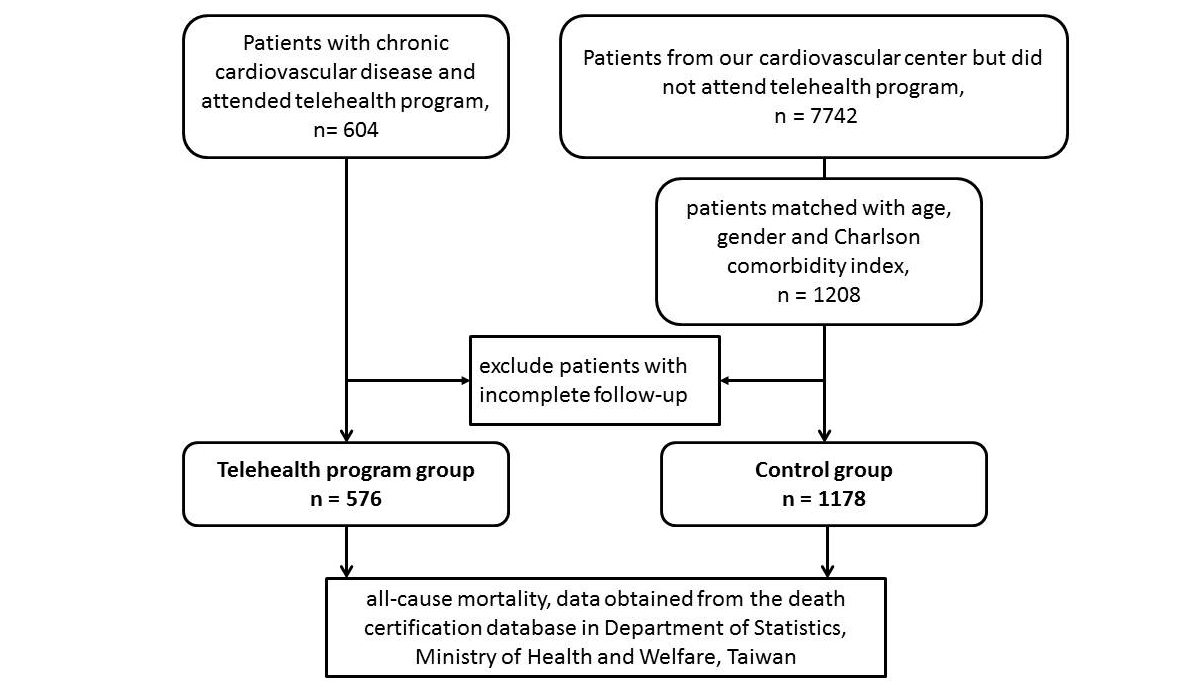
The flow diagram of patient enrollment.

The study was conducted from December 2009 to April 2013 at the Telehealth Center of the hospital, and conducted by the Taiwan ELEctroHEALTH study group (TELEHEALTH study group). The original study method has been described previously [8]. The flow diagram of patient enrollment is shown in Figure 1. Briefly, patients older than 20 years with chronic CVD receiving the telehealth program at our telehealth center were enrolled as the study group. The decision for receiving the telehealth program was depended on patients and/or their caregivers. Chronic CVD included coronary artery disease (CAD), myocardial infarction, heart failure, peripheral artery disease (PAD), stroke, and hypertension. The control group included participants who visited our cardiovascular center during the same period, but did not participate in the telehealth care program (received usual care only). The exclusion criteria in this study (for both telehealth group and control group) included: (1) younger than 20 years, (2) patients without any one of chronic cardiovascular diseases, and (3) patients not followed in our hospital. After excluding ineligible patients, a total of 576 cases and 1178 controls were enrolled in this study.

### Telehealth Care Program

The fourth-generation telehealth program at our telehealth center is a synchronized and integrated remote management program for chronic diseases. The Internet-based platform was developed by the Graduate Institute of Biomedical Electronics and Bioinformatics, National Taiwan University, Taiwan. The details of this program have been reported previously [8]. Briefly, this telehealth program provides the following services: (1) biometric data, including single-lead electrocardiography, blood pressure, heart rate, and oximetry, are transferred from patients to our telehealth center daily and on-demand; (2) nurse case managers telephone patients daily and on-demand for communication and health promotion; (3) full-time nurse case managers and cardiologists are in charge of care 24 hours a day; and (4) long-term medication and management are discussed with the patients’ primary care physician after acute events. This telehealth program bridges acute and home care and emphasizes education, prevention, and early detection of clinical deterioration.

### Usual Care

Patients in the control group received the usual care provided by the primary care physicians at our cardiovascular center according to updated guidelines including, but not limited to, the American Heart Association’s guidelines for lifestyle modification and primary prevention to reduce cardiovascular risk, guidelines for the management of stable ischemic heart disease, and the American Diabetes Association’s guidelines for the management of diabetes. Patients made routine outpatient department visits (once every 3 months) to their primary care physicians. There was no contact between the telehealth center and patients receiving usual care.

### Data Collection

All demographic and clinical data were obtained from the electronic database of the hospital. The diagnosis of a chronic disease was based on the electronic database. The discharge diagnosis was used if the outpatient and discharge diagnoses disagreed.

The primary outcome of this study was all-cause mortality. Mortality data were obtained from the death certification database in the Department of Statistics, Ministry of Health and Welfare, Taiwan.

### Statistical Analysis

Statistical analysis was performed using the R version 2.14.0 software (R Foundation for Statistical Computing, Vienna, Austria). A two-sided *P* value ≤.05 was considered statistically significant. The continuous variables are presented as mean (SD), whereas categorical variables are presented as frequency and percentage. In univariate analysis, the potential predictive factors of all-cause mortality were examined by the chi-square test, Fisher exact test, two-sample *t* test, or Wilcoxon rank sum test as appropriate for the data type. Next, multivariate analysis was conducted by fitting the Cox proportional hazards model to estimate the adjusted effects of predictive factors on time to all-cause mortality.

To ensure the quality of analysis, the model-fitting techniques for (1) variable selection, (2) goodness-of-fit assessment, and (3) regression diagnostics were used in our regression analyses. Specifically, the stepwise variable selection procedure (with iterations between the forward and backward steps) was applied to obtain the candidate final Cox proportional hazards model. All the univariate significant and nonsignificant relevant covariates listed in Table 1 of Ho et al [8] (eg, age, sex, and comorbidities) and some of their interactions were put on the variable list to be selected. The significance levels for entry and for stay were set to .15 to be conservative. Simple and multiple generalized additive models (GAMs) of the binary response (1=dead vs 0=alive) were fitted to detect nonlinear effects of continuous covariates and identify appropriate cut-off point(s) for discretizing them, if necessary, during the stepwise variable selection procedure. Then, with the aid of substantive knowledge, the best candidate final Cox proportional hazards model was identified manually by dropping the covariates with *P*>.05 one at a time until all regression coefficients were significantly different from zero. Any discrepancy between the results of the univariate analysis and multivariate analysis was likely due to the confounding effects of uncontrolled covariates in univariate analysis or the masking effects of intermediate variables (or mediators) in the multivariate analysis.

Finally, the statistical tools of regression diagnostics for verification of proportional hazards assumption, residual analysis, detection of influential cases, and check of multicollinearity were applied to discover any model or data problems. The required proportional hazards assumption was tested based on the scaled Schoenfeld residuals. We added interaction terms between time (days) and the covariates that violated the proportional hazards assumption into the Cox proportional hazards model to examine their time-varying effects on all-cause mortality in the stepwise variable selection procedure. Technically, the original wide-form survival data were reconstructed into a long-form data structure using the so-called counting process style of input for fitting such Cox regression models. Numbers needed to treat was estimated based on the hazard ratio of telehealth program [10].

## Results

### Descriptive Statistics

A total of 1754 patients (576 in the telehealth group and 1178 in the control group) were enrolled in this study (Figure 1). The baseline characteristics were reported previously. Briefly, the mean age was 64.5 (SD 16.0) years and 61.17% (1073/1754) were male. At baseline, age, sex, and Charlson comorbidity index (1.35 in telehealth group vs 1.21 in the control group, *P*=.07) were comparable between the two groups. In the telehealth group, however, there were more patients with CAD (243/576, 42.2% vs 392/1178, 33.28% in telehealth group vs control group, respectively), heart failure (112/576, 19.4% vs 186/1178, 15.79%), stroke (71/576, 12.3% vs 110/1178, 9.34%), dementia, chronic obstructive pulmonary disease, diabetes, and peptic ulcer disease (Table 1 in [8]). The median follow-up time was 566 (IQR 349-807) days for the telehealth group and 1074 (IQR 524-1280) days for the control group. During the follow-up period, there were less hospitalizations (mean 0.05, SD 0.12 per month vs mean 0.11, SD 0.21 per month, *P*<.001) and emergency department visits (mean 0.06, SD 0.13 per month vs mean 0.09, SD 0.23 per month, *P*<.001) in the telehealth group compared with the control group. The outpatient visit times were comparable between the two study groups (mean 1.57, SD 1.12 per month vs mean 1.66, SD 1.78 per month, *P*=.75).

### Survival Outcome

**Figure 2:**
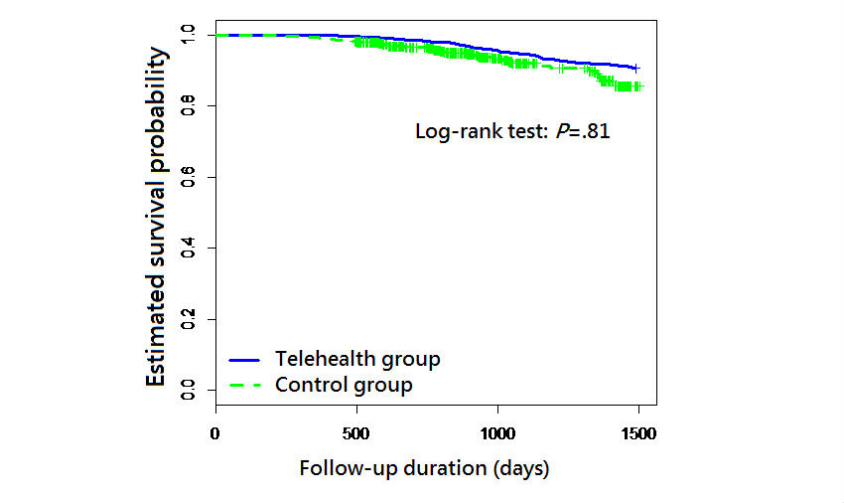
The Kaplan-Meier estimate of the survival curves for all-cause mortality.

There were 53/576 (9.3%) deaths in the telehealth group and 136/1178 (11.50%) deaths in the control group. The Kaplan-Meier survival curve is shown in Figure 2. The estimated survival curves of the two study groups were similar during the follow-up period (log rank test: *P*=.81). Because the baseline comorbidities were heterogeneous and the follow-up durations differed between the two study groups, Cox proportional hazards analyses were performed. A proportional hazards assumption was tested for each variable using the Schoenfeld residuals test. The result showed the proportional hazards assumption was violated for the telehealth program (Table 1). The time-varying telehealth program effect was then applied to the Cox regression model to examine its effect on mortality. The time-varying Cox regression analysis revealed a hazard ratio (HR) of 0.866 (95% CI 0.837-0.896, *P*<.001) for the telehealth program on all-cause mortality. The time-dependent interaction term in this Cox regression analysis indicated that the beneficial effect of the telehealth program on the HR would increase over time. Thus, the estimated numbers needed to treat for the telehealth program to prevent one death at 1, 2, and 3 years were 55.6, 40.5, and 27.7, respectively (Table 2).

**Table 1:** The estimated adjusted hazard ratios for the clinical predictors of all-cause mortality obtained from a multivariate Cox regression model.

Clinical predictors	HR (95% CI)	*P*
Age (years)	1.019 (1.018-1.021)	<.001
Age > 69.809 (years)	1.890 (1.810-1.974)	<.001
Age > 69.809 (years) × telehealth program	0.837 (0.788-0.889)	<.001
Male	1.152 (1.125-1.179)	<.001
Telehealth program	0.866 (0.810-0.926)	<.001
Telehealth program × time-to-mortality (days)	0.9997 (0.9996-0.9998)	<.001
Heart failure	1.941 (1.891-1.992)	<.001
Myocardial infarction	1.097 (1.051-1.146)	<.001
Coronary artery disease	0.843 (0.823-0.865)	<.001
Peripheral arterial disease	0.824 (0.786-0.864)	<.001
Dementia	1.204 (1.115-1.301)	<.001
Chronic obstructive pulmonary disease	1.147 (1.107-1.189)	<.001
Connective tissue disease	1.949 (1.784-2.130)	<.001
Liver disease, mild	1.263 (1.187-1.344)	<.001
Liver disease, moderate to severe	1.876 (1.663-2.117)	<.001
Diabetes mellitus	1.291 (1.259-1.325)	<.001
Diabetes mellitus with end organ damage	0.775 (0.713-0.843)	<.001
Chronic kidney disease, moderate to severe	2.362 (2.295-2.430)	<.001
Malignancy	3.403 (3.313-3.496)	<.001
Malignancy with metastasis	2.816 (2.644-3.000)	<.001
Peptic ulcer disease	1.260 (1.196-1.328)	<.001

**Table 2:** The estimated numbers needed to treat at various times after telehealth program (based on the hazard ratio of the telehealth program).

Time from treatment	Number needed to treat	95% CI
1 year	55.6	43.2-75.7
2 years	40.5	31.8-54.7
3 years	27.7	22.0-37.0

## Discussion

### Principal Results

The major finding of this study is that our fourth-generation telehealth program was associated with lower all-cause mortality after controlling for multiple comorbidities among patients with chronic cardiovascular disease. Prior studies on the effects of telehealth programs on all-cause mortality among patients with chronic diseases revealed mixed results [11-13]. This study demonstrated the clear benefits of a newer generation of telehealth care among a population with a broader range of cardiovascular diseases during a longer follow-up duration. In addition, our prior study demonstrated that a fourth-generation telehealth program was independently associated with lower numbers of emergency department visits, hospitalizations, and medical costs (US $587.60 vs US $1163.60 per month in the telehealth and control groups, respectively, *P*<.001) [8]. Based on this result and on our previous reports, our data show a fourth-generation telehealth program is cost-effective and lifesaving.

### Comparison With Previous Work

Among patients with chronic heart failure, the beneficial effect of telehealth care on all-cause mortality has been repeatedly demonstrated in studies [3,4]. The effect of telehealth care among patients with a broader range of chronic comorbidities, however, is still controversial. In the Whole System Demonstrator (WSD) study including 3230 participants with diabetes, chronic obstructive pulmonary disease, or heart failure, a second-generation telehealth program was related to lower all-cause mortality [12]. In a recent study conducted by the Mayo Clinic on 205 frail, older adults with a broader range of chronic diseases, a second generation of telemonitoring resulted in higher mortality compared with usual care [6]. Several factors may contribute to the contradictory results between these two studies and our own studies.

First, the telehealth program has changed over time. Early telemonitoring systems used asynchronous techniques to record and process the collected subjective symptoms and physiological data. A response to the data collected could take more than 1 day during the off hours. This newer system uses a synchronous technique to respond to the information received to act in a timely manner. Through quick communication, more accurate diagnoses and decisions can be made. Second, the age of the study participants differs. The mean ages in the studies were 80.3 years in the Mayo study, 70.3 years in the WSD study, and 64.5 years in our study. The potential barriers for elderly participants to effectively use a telehealth program include a lack of skill to operate a new device, psychological resistance to new technology, and the presence of more comorbidities. Third, the comorbidity profiles also differed. The reported chronic comorbidities were as follows: a mean Charlson score of 2.9 in the Mayo study, a mean number of chronic conditions of 1.8 in the WSD study, and a mean Charlson score of 1.3 in our study. In a meta-analysis on the effectiveness of telemonitoring for four chronic conditions, the results were more consistent for cardiac and pulmonary conditions compared with diabetes and hypertension [14]. Whether or not telehealth programs are effective for all types of chronic conditions is still unknown. The difference in the telemonitoring technology, age, and comorbidities may contribute to the different results from the three studies. These differences warrant further investigation in the future.

Our study found that the effect of the telehealth program on mortality was time dependent; namely, that the beneficial effect increased with longer follow-up durations. Although an increasing benefit on mortality has not previously been reported, an increasing benefit on other surrogate endpoints has been noted previously. In a study of telemedicine conducted with elderly Medicare patients, glycated hemoglobin (HbA1c) and blood pressure levels were lower after the follow-up for 5 years. The difference between the telemedicine and control groups increased further at 5 years [15]. It is plausible that the continuous improvement in the control of chronic conditions, including hypertension and hyperglycemia, gradually led to a benefit in mortality. Moreover, continuous education via daily telephone communication could have improved the knowledge and techniques used by the caregivers. Through repeated discussion of the condition, caregivers learn to better manage the acute exacerbation. This may also have contributed to further improvements in outcomes during the follow-up period.

The paradoxical associations of CAD and PAD with lower all-cause mortality found in our data were unexpected. Plausible explanations of these survival paradoxes included chance or unmeasured residual confounding factors, especially effective treatment for CAD and PAD. In a recent large cohort of 102,023 patients with stable CAD, a history of percutaneous coronary intervention or coronary artery bypass graft in the past 6 months were associated with hazard ratios of 0.651 and 0.516, respectively, on all-cause mortality [16]. It is possible that our results reflect the unmeasured treatment effect of these two diseases. In addition, the patients in our telehealth group had higher rates of CAD and PAD compared with those in the control group. Although we had evaluated the collinearity between different predictors with the variance inflation factor, it is still possible that the paradoxical protective effect reflected the protective effect of the telehealth program, rather than the effect of CAD or PAD.

### Limitations

Our study has some limitations. First, because our study was not a randomized study, unmeasured confounding factors might have influenced the results. Second, because our study compared the whole service program and not any single monitoring device or physiological parameter, the effect of each device or parameter on mortality cannot be assessed separately. Third, because all-cause mortality instead of mortality from specific causes was used in this study, we cannot draw a definite conclusion regarding the effects of telehealth monitoring on specific causes of death. Finally, the numbers needed to treat is estimated based on the hazard ratio of the telehealth program. This may lead to overestimating the numbers needed to treat.

### Conclusions

Our data suggest that a fourth-generation telehealth program is independently associated with lower all-cause mortality among patients with chronic CVD and multiple comorbidities.

## Abbreviations

CAD: coronary artery disease
CVD: cardiovascular disease
GAM: generalized additive models
HR: hazard ratio
PAD: peripheral artery disease
WSD: Whole System Demonstrator
